# A Novel Protocol Using Small-Scale Spray-Drying for the Efficient Screening of Solid Dispersions in Early Drug Development and Formulation, as a Straight Pathway from Screening to Manufacturing Stages

**DOI:** 10.3390/ph11030081

**Published:** 2018-08-27

**Authors:** Aymeric Ousset, Rosanna Chirico, Florent Robin, Martin Alexander Schubert, Pascal Somville, Kalliopi Dodou

**Affiliations:** 1 School of Pharmacy and Pharmaceutical Sciences, Faculty of Health Sciences and Wellbeing, University of Sunderland, Sunderland SR13SD, UK; aymeric.ousset@gmail.com; 2 UCB Pharma S.A., Product Development, B-1420 Braine l’Alleud, Belgium; Rosana.Chirico@ucb.com (R.C.); Florent.Robin@ucb.com (F.R.); Martin-Alexander.Schubert@gmx.net (M.A.S.); Pascal.Somville@ucb.com (P.S.)

**Keywords:** amorphous solid dispersions, screening, spray-dryer, downscaling, polymers, miscibility, dissolution, supersaturation, stability

## Abstract

This work describes a novel screening strategy that implements small-scale spray-drying in early development of binary amorphous solid dispersions (ASDs). The proposed methodology consists of a three-stage decision protocol in which small batches (20–100 mg) of spray-dried solid dispersions (SDSDs) are evaluated in terms of drug–polymer miscibility, physical stability and dissolution performance in bio-predictive conditions. The objectives are to select the adequate carrier and drug-loading (DL) for the manufacturing of robust SDSD; and the appropriate stabilizer dissolved in the liquid vehicle of SDSD suspensions, which constitutes the common dosage form used during non-clinical studies. This methodology was verified with CDP146, a poorly water soluble (<2 µg/mL) API combined with four enteric polymers and four stabilizers. CDP146/HPMCAS-LF 40:60 (*w*/*w*) and 10% (*w*/*v*) PVPVA were identified as the lead SDSD and the best performing stabilizer, respectively. Lead SDSD suspensions (1–50 mg/mL) were found to preserve complete amorphous state during 8 h and maintain supersaturation in simulated rat intestinal fluids during the absorption window. Therefore, the implementation of spray-drying as a small-scale screening approach allowed maximizing screening effectiveness with respect to very limited API amounts (735 mg) and time resources (9 days), while removing transfer steps between screening and manufacturing phases.

## 1. Introduction

Non-clinical testing is a mandatory step of drug development that aims to evaluate the pharmacodynamics, pharmacokinetics and toxicity profiles of new chemical entities (NCEs) so that safe initial dose level can be identified for the first human exposure [1]. In general, liquid suspension formulations represent the most common dosage form used during non-clinical studies because of their ease of preparation and applicability to the vast majority of non-clinical species [2,3]. Suspension formulations consist of the dispersion of drug solid particles throughout an aqueous medium containing additional excipients, typically emulsifying, suspending or ionizing agents as well as surfactants and solvents [4]. This standard non-clinical formulation is generally administrated to animals via oral gavage. Therefore, oral non-clinical formulations such as liquid suspensions differ from conventional oral dosage forms used during clinical trials since tablets or capsules are not adapted for the dosing of most non-clinical species [5].

However, as the number of NCEs with poorly water soluble properties is continuously increasing during drug discovery phase, the conduction of non-clinical studies becomes challenging [3,6]. In this regard, the use of amorphous solid dispersions (ASD) has become a common strategy to tackle low solubility and improve absorption of the active pharmaceutical ingredient (API) in early drug development and formulation [7]. One of the major benefits of using ASD dosed as suspension formulation in comparison to the crystalline drug is the capability of producing adequate exposure of the API during non-clinical studies [8]. The strategy of solid dispersions consists of the drug amorphization and its dispersion into a polymeric carrier [9]. Spray-drying is considered as the solvent based process of reference for the manufacturing of solid dispersions in the pharmaceutical industry [10]. The key expectations regarding the development of a solid dispersion are the successful manufacturing of the amorphous form of the drug, its stability during the shelf life of the product and its capacity to maintain supersaturation during dissolution test [11].

Because, the selection of the appropriate polymer and drug-loading (DL) prior to the manufacturing of spray-dried solid dispersions (SDSDs) should be addressed in the first stage of the product’s development, the use of miniaturized screening methodologies have gained a large interest in the pharmaceutical industry. More specifically, solvent casting screening has been particularly used in the industrial sector due to its capacity to operate in an automated mode allowing the testing of a maximum number of carriers while using a minimum amount of drug product [12,13].

However, the effect of the preparation method on the properties of screened ASDs and, more generally, on the outcome of the screening is often underestimated [14]. The fact that standard screening methodologies e.g., solvent casting and quench cooling are not representative of the operating mode and process conditions of regular spray-dryer, increases the risk to generate ‘false negative and positive’ results [15,16]. These samples are known to display different properties in terms of drug–polymer miscibility, glass transition temperature (T_g_), physical stability, inter-components interactions and dissolution kinetics between screening and manufacturing scale [15,17,18,19]. This limits the prediction accuracy of conventional screening approaches because API-polymer systems can be abandoned prematurely during screening phases or can present limited potential when manufactured. The frequency of conventional screening methods to generate ‘false negatives/positives’ is not evaluated in a systematic way and increases the risk of inappropriate carrier selection. Finally, transfer from screening phases to laboratory scale production generally requires time and resources as a set of new experiments need to be carried out in order to finetune optimal DL and determine appropriate processing conditions [8].

Considering the increasing number of poorly soluble NCEs requiring formulation to ASDs, and the drawbacks of the current screening processes, there is a need for the development of a more reliable screening method that would also be time-efficient and simple enough to be used on a routine basis by the industry. In this regard, this work aims to propose a novel screening strategy at preclinical scale that integrates laboratory spray-drying throughout all phases of API development from the screening phases to the first SDSD batch production to support GLP non-clinical studies. The novelty of this work is to overcome preconceived ideas about the use of laboratory spray-drying at small-scale and demonstrates its suitability to operate in the case of resource limited compounds as a practical solution for pharmaceutical scientists

The present paper describes a new step-wise strategy for the (i) the screening of binary SDSD in early drug and formulation, and (ii) the development of SDSD dosed as non-clinical suspension formulation for oral administration. This novel screening approach was applied for CDP146: a poorly water soluble API from the UCB pipeline that is a candidate for the treatment of epilepsy. Firstly, the screening of appropriate polymer and optimal DL for the manufacturing of the lead SDSD that combines the best performance in terms of physical stability and solubility improvement was conducted during the first two stages of the proposed protocol. To do that the performance of API-polymer combinations made of four polymeric carriers (HPMCP HP50, HPMCAS-LF, Eudragit L100 and Eudragit L100-55) was examined. In the present study, the selection of enteric polymers is explained by their particular interest during non-clinical studies due to their ability to protect drug from recrystallization in gastric fluid and delay supersaturation until the drug reaches the upper small intestine so that absorption is maximized [20,21]. Furthermore, typical DL used during non-clinical studies is usually in the range 25–40% (*w*/*w*) [8,18]. Thus, a DL of 40% (*w*/*w*) was initially tested as it would allow reaching high doses (of up to 1 g/kg of drug administrated per day) in toxicology dose escalation studies while limiting the amount of excipients administrated to the animals with regard to toxicity and tolerability. From experience, DL higher than 40% (*w*/*w*) can negatively impact the dissolution performance of ASDs by acting as a driving force for recrystallization. Secondly, the previously identified lead SDSD was prepared as liquid oral suspension formulation. The term ‘oral formulation’ refers to the preparation of SDSD suspended in the vehicle made of an aqueous medium containing HPC-SSL as standard suspending agent and one additional stabilizer. Although, the role of the carrier in ASD blend is to prevent drug from recrystallization in the solid state and during dissolution, the high doses usually tested during non-clinical studies generally require the use of an additional stabilizer dissolved in the liquid vehicle to ensure no physical change of the drug in the suspension that can negatively impact exposure. Accordingly, four conventional crystallization inhibitor agents including SDS, HPMC, Vitamin ETPGS and PVPVA have been screened. The choice of the above stabilizers and their concentration in the oral formulation vehicle have been carefully selected to minimize potential adverse effects and toxicity to the tested animals [4].

## 2. Material and Methods

### 2.1. Materials

**Figure 1:**
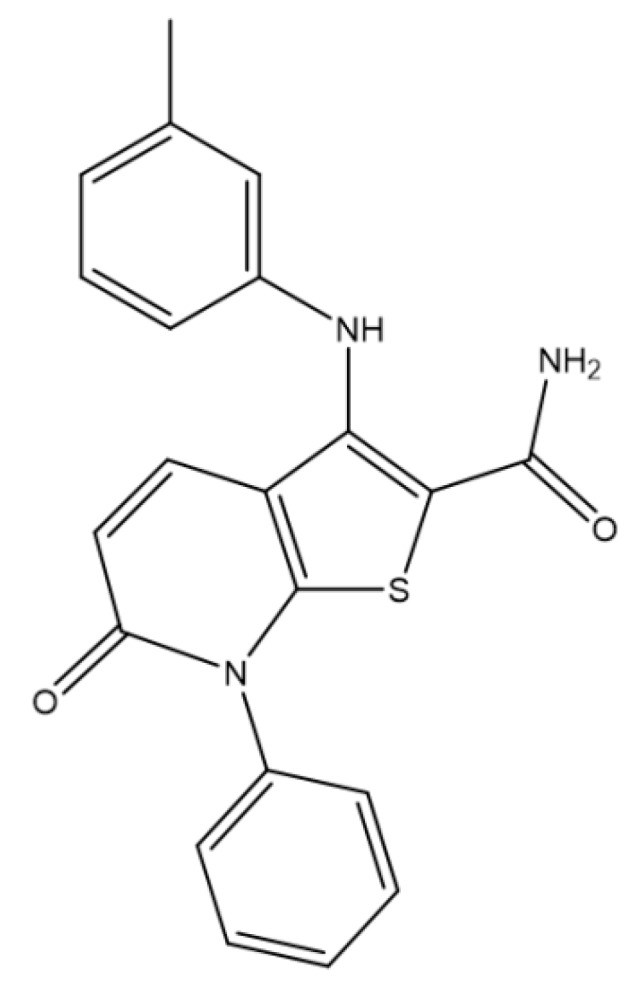
Chemical structure of CDP146.

Crystalline CDP146 was obtained from UCB Pharma, Product Development department (Braine l’Alleud, Belgium). The chemical structure of CDP146 is depicted in Figure 1. This compound has a molecular weight of 375.45 g/mol, a T_g_ of 95 °C, a melting temperature (T_m_) of 198.1 °C. The API solubility in water was determined at 37 °C after 24 h under stirring (250 rpm) with drug content in excess within the aqueous medium. Following this procedure, the drug solubility was found to be below 2 µg/mL from pH 1.2 to pH 10.0.

Four enteric polymers were evaluated as potential carriers for ASD: hydroxypropylmethylcellulose phthalate (HPMCP HP50) and hydroxypropylmethylcellulose acetate succinate fine grade (HPMCAS-LF) obtained from Shin-Etsu (Tokyo, Japan), copolymer of methacrylic acid and methyl methacrylate 1:1 (Eudragit L100) and copolymer of methacrylic acid and ethyl acrylate copolymer 1:1 (Eudragit L100-55) purchased from Evonik (Essen, Germany). Table 1 summarizes the physico-chemical and thermal properties of the selected carriers.

Four stabilizers were tested with regard to the stabilization of suspension formulation: copolymer of *N*-vinyl-2-pyrrolidone and vinyl acetate (PVPVA) from Ashland (Covington, KY, USA), sodium dodecyl sulfate (SDS) from Merck (Darmstadt, Germany), hydroxypropylmethylcellulose (HPMC) and vitamin ETPGS from Sigma Aldrich (Saint-Louis, MO, USA). Hydroxypropylcellulose (HPC-SSL) from Nisso (Düsseldorf, Germany) was used for oral formulation preparation. Simulated intestinal fluids (SIF) powder was obtained from Biorelevant.com (London, UK) and used to simulate rat intestinal fluid. All other materials and solvents used were of reagent and analytical grade, respectively.

### 2.2. Methods

#### 2.2.1. Screening Strategy

##### General Considerations

**Figure 2:**
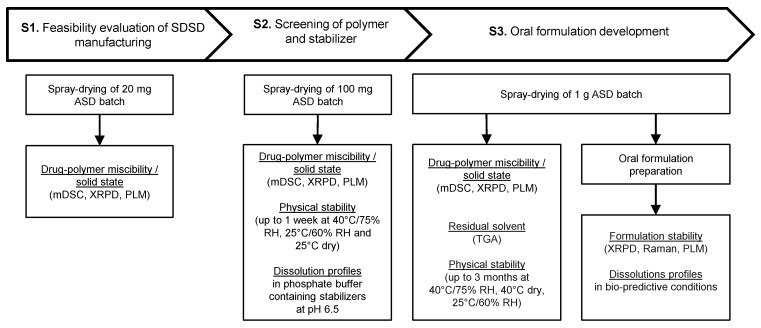
Summary of the experimental protocol applied in the proposed screening strategy.

**Figure 3:**
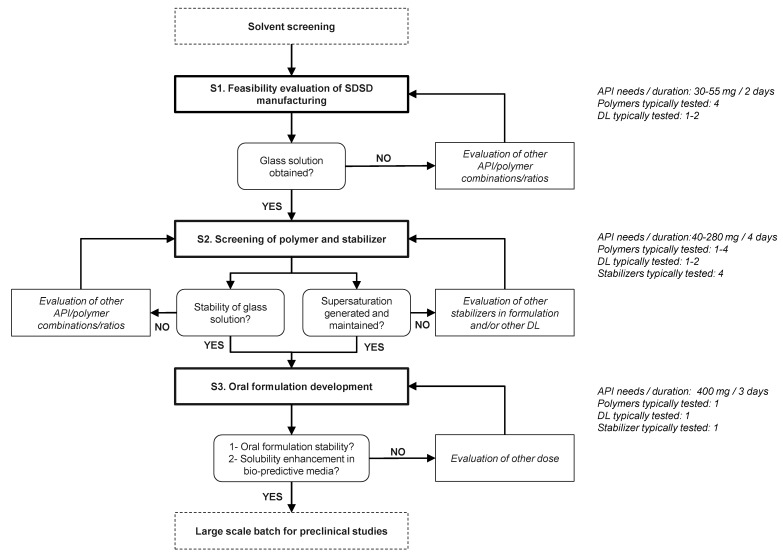
Flow chart representation of the three-stages’ decisional screening strategy.

Figure 2 and Figure 3 describe the experimental protocol summary and the flow chart representation of the proposed screening strategy, respectively. As seen in Figure 3, the proposed screening protocol uses a three-stage decision tree including “Feasibility evaluation of SDSD manufacturing”, “Screening of polymer and stabilizer” and “Oral formulation development”. API and time consumption have been estimated while considering the screening of four polymers at two DLs (e.g., 30 and 40% (*w*/*w*)) for the manufacturing of binary SDSD, and four stabilizers dissolved in the liquid vehicle of the oral non-clinical formulation.

- As a preliminary step of screening protocol, a common solvent or binary solvent mixture of interest that allows dissolving both drug and polymer, needs to be identified. A solute concentration higher than 2% (*w*/*v*) is defined as acceptance criteria for spray-drying development to achieve a reasonable yield, process time and solvent consumption to comply with HSE considerations [8,23].
- In the first Stage (S1), the potential of screened carriers was evaluated based on their ability to generate glass solutions by spray-drying. The evaluation of drug–polymer miscibility in the early stage of drug development is known to offer a reliable assessment of the ASD potential [24,25,26]. Specifically, the formation of glass solution system where amorphous drug is molecularly dispersed in the carrier, combines the best performance in terms of physical stability and solubility improvement [27]. On the contrary, semi-crystalline and phase-separated ASDs are known to provide limited potential of solubility enhancement and higher tendency for drug recrystallization during both dissolution and upon storage [28,29]. Commonly used excipients for the preparation of solid dispersions include cellulose, polyvinlylpyrrolidone, poloxamer, polyethylene glycol or polymethacrylate derivatives [30]. These polymers are recognized as “generally regarded as safe” (GRAS) excipients. Additional criteria such as T_g_, hygroscopicity, solubility in organic solvents, viscosifying properties, pH of hydration in water and solid solution capacity need to be considered regarding the carrier selection for the manufacturing of solid dispersions by spray-drying [31].
- In the second Stage (S2), the physical stability of API-polymer systems identified as glass solutions was assessed up to one week. In parallel, the dissolution properties of these ASDs was examined with and without stabilizers dissolved in aqueous medium. Therefore, the influence of the stabilizer in the dosing vehicle of the oral formulation and its ability to maintain drug supersaturation and parachute effect during dissolution tests were investigated. The use of stabilizer in the dosing vehicle of suspensions allows converting the API into suitable dosage form for administration to non-clinical species. The wide variety of stabilizers commonly used during non-clinical studies allows overcoming the diversity of molecule specific exposure limitations so that the formulation maintains stability, homogeneity and dosability within the range of doses tested [5].
- In the last Stage (S3), the long-term stability of the lead SDSD identified during Stage 2 was investigated for up to 3 months. Moreover, the lead SDSD was prepared as non-clinical suspension formulation in the vehicle containing the stabilizer of interest. Then, the oral formulation was prepared at various doses generally tested during non-clinical studies. Its stability prior to administration and dissolution performance in bio-predictive conditions were assessed.

##### Application to CDP146 ASD Screening

● Feasibility evaluation of SDSD manufacturing (Stage 1)

Productions of 20 mg batches of 40:60 (*w*/*w*) CDP146 ASDs with HPMCP HP50, HPMCAS-LF, Eudragit L100 and Eudragit L100-55 were performed using the laboratory scale ProCept 4M8-TriX spray-dryer (Zelzate, Belgium). This equipment was selected due to its capability to operate with feed solution volume as low as 0.5 mL and up to 24 L/8 h [32,33]. API-polymer solutions were prepared in Dichloromethane (DCM)/Ethanol (EtOH) 2:1 (*v*/*v*) at 50 mg/mL. The feed solution was pumped to the nozzle via a peristaltic pump Watson Marlow 530S (Falmouth, Cornwall, UK) and adjusted at 1 g/min. Atomization of the feed solution into fine droplets was achieved using a bifluid nozzle with a diameter of 1.2 mm and an atomizing air pressure of 1.50 bars. Solvent evaporation was performed by using a drying gas airflow of 0.30 m^3^/min at an inlet temperature of 60 °C. A lateral cooling airflow of 100 L/min was applied to transfer powder from the bottom of the drying chamber to the small cyclone (height/diameter of 210 mm/40 mm) where particle separation occurs. The present manufacturing conditions have been specifically optimized to maximize the yield of small-scale batches of solid dispersions. In addition, a customized 3D printed funnel has been particularly developed to allow powder collection into a standard aluminum pan (TA Instruments, Leatherhead, UK) used for modulated differential scanning calorimetry (mDSC) analysis. This powder collection system allows reducing material loss during powder handling and eases the transfer for subsequent analytical characterization. After processing, the collected powders were stored in a vacuum oven for 48 h before analysis. Duplicate SDSD productions were conducted per API-polymer combination to assess reproducibility: one batch was analyzed by mDSC while the repeated sample was analyzed by X-ray powder diffraction (XRPD).

● Screening of polymer and stabilizer (Stage 2)

100 mg batches of 40:60 (*w*/*w*) CDP146 solid dispersions identified as ‘glass solutions’ during Stage 1 were produced by spray-drying. The same process and formulation conditions used for the 20 mg batch productions were maintained. In this particular case, spray-dried material was collected in a 2 mL glass vial (Waters, Saint-Quentin-en-Yvelines, France) connected to the 3D printed system. SDSDs were analyzed by mDSC and XRPD, respectively, to confirm up-scaling robustness and comparability between Stage 1 and Stage 2 with regard to miscibility and solid state. The physical stability of produced solid dispersions was assessed after one week upon storage under both stress and ambient storage conditions. About 10 mg of spray-dried powder were placed in an incubator at 40 °C/75% Relative humidity (RH), 25 °C/60% RH and 25 °C dry storage conditions. The aforementioned storage conditions were selected in line with the ICHQ1A(R2) guidelines. After one week, SDSDs were analyzed by mDSC, XRPD and polarized light microscopy (PLM) to detect the presence of potential drug crystals formed upon storage.

An accurate weight of 2.5 mg of SDSD (equivalent to 1 mg of API) was dosed into 1 mL of dissolution medium consisting of 50 mM phosphate buffer at pH 6.5 with stabilizers. Five dissolution media with different stabilizer compositions were tested: Vehicle A (no stabilizers), Vehicle B (1% (*w*/*v*) HPMC), Vehicle C (0.2% (*w*/*v*) SDS), Vehicle D (1% (*w*/*v*) vitamin ETPGS) and Vehicle E (10% (*w*/*v*) PVPVA). The dissolution profile of pure crystalline CDP146 was obtained in each dissolution medium so that the solubility improvement (%) of screened SDSD can be evaluated. The aforementioned dissolution conditions represent non-sink conditions with respect to the crystalline drug so that the ability of screened ASD to generate and maintain supersaturation can be assessed during the duration of the test [34]. Dissolution tests were carried out manually in 10 mL glass tube (VWR, Heverlee, Belgium). Mixtures were maintained at 37 °C under magnetic stirring at 250 rpm using Thermo Mixer C unit (Eppendorf, Hamburg, Germany). Samples of 100 μL were collected after 5, 30, 60, 120, 180, 240, 360 and 1440 min and transferred into 0.45 µm ultrafree filter units (Merck Millipore, Burlington, MA, USA). Samples were centrifugated at 8000 rpm during 2 min. The filtrate was pipetted, properly diluted in H_2_O/Acetonitrile (ACN) 1/1 (*v*/*v*) and analyzed in High performance liquid chromatography (HPLC).

● Oral formulation development (Stage 3)

1 g of lead ASD of CDP146 was produced by spray-drying using the laboratory scale ProCept 4M8-TriX spray-dryer (Zelzate, Belgium). Drug–polymer solutions were prepared in the binary solvent mixture of interest DCM/EtOH 2:1 (*v*/*v*) at 50 mg/mL. The feed solution flow rate was adjusted at 5 g/min. An atomizing air pressure of 0.65 bars was applied to a 1.2 mm bifluid nozzle to create a spray. The drying gas airflow was set at 0.35 m^3^/min and maintained at 65 °C. The lateral cooling air was kept constant at 100 L/min and dried particles were separated from the exhaust air within the medium cyclone (height/diameter of 242 mm/60 mm). After processing, the spray-dried material was stored in a vacuum oven for 48 h before analysis to eliminate the last traces of residual solvent.

The solid state and miscibility of spray-dried material was analyzed by XRPD, PLM and mDSC, respectively, to confirm the results obtained during previous screening steps. Residual solvent analysis was carried by Thermogravimetric analysis (TGA). The long term stability of the lead SDSD of CDP146 was investigated up to 3 months at 40 °C/75% RH, 40 °C dry storage conditions and 25 °C/60% RH. Solid state evolution and residual solvent content were determined after 2 weeks, 1, 2 and 3 months. This would help to gain insight into the shelf life of the formulated drug.

In order to obtain ASD suspension, SDSD powder was dispensed in a 100 mM citrate buffer (pH 4) vehicle containing 1% (*w*/*v*) HPC-SSL, 0.1% (*w*/*v*) antifoam and the stabilizer agent of interest identified during the second step of the screening procedure. Oral liquid formulation was prepared at various API concentrations of 1, 10, 30 and 50 mg/mL. Powder was accurately weighed into 4 mL Nalgene polypropylene vial (Thermo Fischer Scientific, Waltham, MA, USA), and half of the required amount of vehicle was added. The mixture was manually mixed during one minute in order to wet all solid particles. Then, the rest of the vehicle was poured into the mixture and the formulation was magnetically stirred at 350 rpm for at least 30 min to remove the presence of air bubbles in the mixture and homogenize the suspension. The formulation stability and homogeneity was determined by XRPD, PLM and Raman spectroscopy up to 8 h corresponding to a working day, under magnetic stirring at 350 rpm.

Dissolution profiles of CDP146 suspension formulation were obtained at various doses in bio-predictive conditions representative for the rat species. First, 2 mL of freshly prepared oral formulation was pipetted into a 10 mL glass tube. 2.4 mL of a medium containing HCl and NaCl (6.19 g/L) at pH 3.2, used to simulate the gastric medium of rat was added to the mixture. The dissolution was carried out at 37 °C under magnetic stirring at 250 rpm using Thermo Mixer C unit (Eppendorf, Hamburg, Germany). Temperature and magnetic stirring were maintained constant during the entire dissolution tests. Samplings of 100 μL were taken after 1, 5, 10, 15 and 30 min. After 30 min of dissolution in simulated rat gastric fluid, 1 mL of the mixture was transferred and diluted with 1 mL of 100 mM phosphate buffer at pH 5.0 containing SIF powder (35.6 mM). This vehicle was used to simulate the composition of the first compartment of rat intestinal fluid. This procedure was repeated twice with the second and third compartment of rat intestinal fluids that consist of 100 mM phosphate buffer at pH 5.0 containing SIF powder (14 mM) and 100 mM phosphate buffer at pH 6.0 containing SIF powder (4 mM), respectively. A dilution factor of 2 from the gastric to each intestinal fluid was applied to be representative for physiological conditions. Samplings of 100 μL were taken after 1, 5, 10, 15, 30, 60, 90 and 120 min. All samples were filtered on 0.45 µm ultrafree filter units (Merck Millipore, Burlington, MA, USA) and centrifugated at 8000 rpm during 2 min. Appropriate dilution in H_2_O/ACN 1/1 (*v*/*v*) was performed and API content was determined in HPLC.

#### 2.2.2. Analytical or Characterization Methods

##### Modulated Differential Scanning Calorimetry

The phase behavior and thermal properties of SDSD were analyzed in mDSC using TA Instruments Q1000 calorimeter (TA Instruments, Leatherhead, UK). The chamber was purged with a 50 mL/min flow rate of dry nitrogen. Indium and sapphire disks were used to calibrate the temperature/enthalpy and heat capacity, respectively. The powder was analyzed in non-hermetic standard aluminum pans (TA Instruments, Leatherhead, UK). Samples were heated from 0 °C to 210 °C at 2 °C/min with a modulation of ±1 °C and a period of 40 s. Data was processed using Universal Analysis 2000 software (TA Instruments, Leatherhead, UK): T_g_ was reported as the mid-point of inflection in the step change observed in the reverse heat flow signal while crystallization and melting events were recorded in non-reverse and total heat flows.

##### Thermogravimetric Analysis

TGA experiments were conducted in a TGA Q500 (TA Instruments, Leatherhead, UK) in order to estimate the percentage of residual solvent and moisture content in spray-dried material. The chamber was swept by a 50 mL/min flow rate of dry nitrogen. Samples were set isothermally 5 min at 25 °C and heated to 300 °C at 10 °C/min. Data were processed using the TA instruments software Universal Analysis 2000 software (TA Instruments, Leatherhead, UK).

##### X-ray Powder Diffraction

The solid state of screened solid dispersions was characterized in XRPD. Analyses were performed on X Bruker AXS D8 Advance (Bruker, Karlsruhe, Germany). Monocrystal silicium holders were used during sample preparation. Analyses on powder were carried out over the range 3.5–30° at a scan speed of 2.5 s/step and a step size of 0.02°. Data was processed using Eva DIFFRAC-SUITE software (Bruker, Karlsruhe, Germany).

XRPD analyses were also conducted in order to monitor the crystallization behavior of the oral liquid formulation. Samplings of 200 μL were taken after 0, 4 and 8 h and were subsequently centrifuged at 8000 rpm during 2 min on 0.45 µm ultrafree centrifugal filter units (Merck Millipore, Burlington, MA, USA). The wet solid filtrate was collected from the filter and analyzed in XRPD over the range 3.5–30° at a scan speed of 0.1 s/step and a step size of 0.02°. Then, a second analysis was conducted on the ‘dried’ product to evaluate the potential appearance of crystals after water evaporation of the wet solid filtrate.

##### Polarized Light Microscopy

The observations were performed using a AX10 Zeiss light microscope (Carl Zeiss, Oberkochen, Germany) under polarized light. The samples were observed in the optical resolution ×400. Pictures were collected using Axiocam MRC5 and images were processed using Axiovision 4.0 software (Carl Zeiss, Oberkochen, Germany). The presence of crystallites was determined by the observation of birefractive entities under polarized light.

##### Raman Spectroscopy

Raman spectroscopy was used to monitor the crystallization behavior of ASD suspension formulation. Analyses were conducted on Raman RXN2 Hybrid Analyzer (Kaiser Optical Systems, Ann Arbor, MI, USA) using a 785 nm laser wavelength in reflexion mode. Spectra were acquired by coupling the analyzer with fiber-optic MR probe with an 1/8″ immersion optic. Internal calibration and determination of optimal exposure time were performed prior to analysis. Analyses of both crystalline API and CDP146 ASD suspension in the vehicle of interest were conducted. The immersion optic was inserted in 1 mL of formulation, previously pipetted into a 2 mL HPLC glass vial with PTFE/silicone septa (Waters, Saint-Quentin-en-Yvelines, France). The mixture was magnetically stirred at 350 rpm and spectra were collected every 15 min during 8 h. Measurements were carried out in a black chamber to prevent interference from ambient. The Raman shift of 150–1890 cm^−1^ was examined. Two regions of Raman spectra where crystalline API and freshly prepared ASD suspension were found to display significant differences in peak characteristics, were particularly investigated. Data was obtained with iC Raman software (Kaiser Optical Systems, Ann Arbor, MI, USA) and processed by Matlab R2017b (Mathworks, Natick, MA, USA).

##### HPLC

Determination of CDP146 content during dissolution tests was performed using HPLC coupled with UV detection. Measurements were conducted on an X Bridge C18 column (Waters, Saint-Quentin-en-Yvelines, France) at 45 °C. The injection volume was fixed at 20 μL and the detection was carried out by UV at 305 nm. The analytical method used a gradient mobile phase composed of a mixture of acetate buffer (pH 4.5) and acetonitrile at a flow rate of 0.8 mL/min. Data were processed by Empower 3 chromatography data software (Waters, Saint-Quentin-en-Yvelines, France). Standard solutions of pure CDP146 were prepared in H_2_O/ACN 1:1 (*v*/*v*) to cover a calibration linearity over the concentration range of 1–75 μg/mL.

## 3. Results and Discussion

### 3.1. Feasibility Evaluation of SDSD Manufacturing (Stage 1)

**Figure 4:**
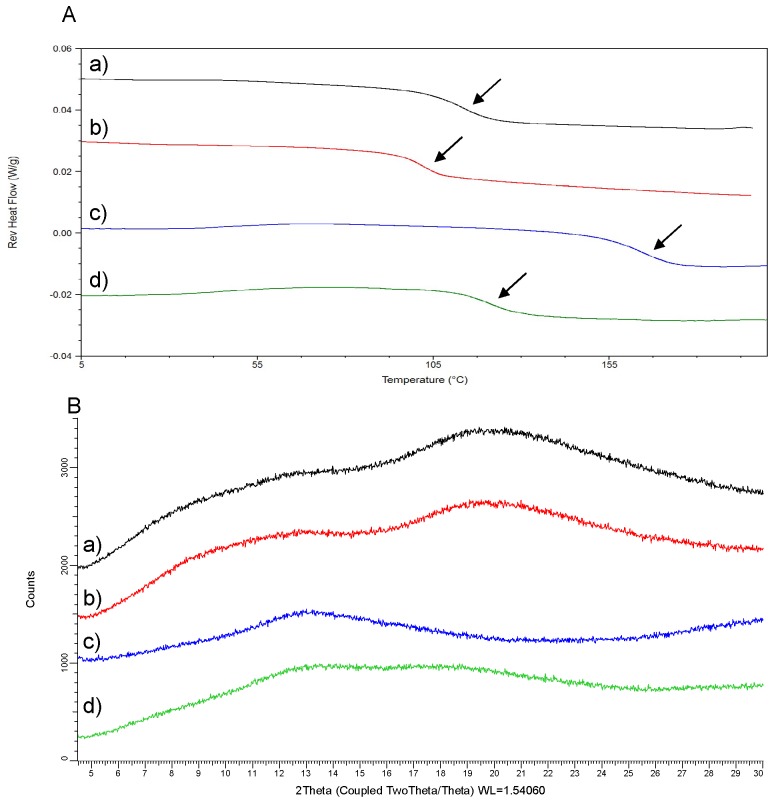
Reverse heat flow signals (**A**) and X-ray powder diffraction (XRPD) patterns (**B**) of 40:60 (*w*/*w*) CDP146 SDSDs (20 mg) made with HPMCP HP50 (a), HPMCAS-LF (b), Eudragit L100 (c) and Eudragit L100-55 (d).

Screened CDP146-polymer systems were produced by spray-drying (20 mg). Yields ranging from 16–28% were obtained so that sufficient material was collected for subsequent solid state characterization. Therefore, API-polymer combinations were evaluated based upon their ability to form glass solutions after processing at a DL of 40% (*w*/*w*). Figure 4 displays the results obtained in the reverse heat flow signal of mDSC and the XRPD patterns of 20 mg ASD batches of CDP146. Glass solutions were obtained for SDSDs of CDP146 made with HPMCP HP50, HPMCAS-LF, Eudragit L100 and Eudragit L100-55. As seen in Figure 4A, the thermograms of these API-polymer systems display a clear T_g_, balanced between the T_g_ of pure components in the blend. No drug melting endotherm was detected in both heat flow and non-reverse signals (data not shown). Additionally, the presence of a large amorphous halo and the absence of Bragg peaks in XRPD pattern confirm the complete amorphization of the drug after processing (Figure 4B). Similar analytic data obtained for the first and the second batch demonstrated that the operating mode of small-scale spray-drying leads to reproducible results. During a ‘real’ screening, the second batch production can be removed i.e., one single batch of screened solid dispersion (20 mg) can be analyzed both in mDSC and XRPD to minimize API consumption.

All tested ASDs were identified as molecularly dispersed glass solutions and can therefore continue in the screening process. At this stage, there is no need to test these API-polymer systems at higher or lower DLs. However, if residual crystallinity is detected for SDSD produced at a DL of 40% (*w*/*w*), additional experiments at lower DL (e.g., 30% (*w*/*w*)) would be needed.

In the current case study, preliminary evaluation of CDP146 SDSD miscibility and solid state was not found to discriminate polymers; this can be explained on the basis that CDP146 has a relatively low tendency for recrystallization. According to the classification of Baird et al. (2010) that aims to categorize the crystallization tendency of APIs from undercooled melts, CDP146 was found to belong to the class III group i.e., molecules that display complete amorphization and no recrystallization during a heating/cooling/heating cycle in DSC [35]. Furthermore, the application of the proposed screening strategy in different UCB pharma development projects has demonstrated that the first screening stage allowed realizing a pre-selection of adequate carriers for the SDSD manufacturing of API with higher tendency for recrystallization (data not shown). Preliminary information regarding the API tendency for recrystallization would allow assessing the suitability for ASD as a formulation principle. Nevertheless, a decision regarding the selection of adequate carrier and DL for the development of SDSD cannot be based on the sole criteria of drug–polymer miscibility evaluation. Conduction of dissolution tests is required to assess the “true” performance of ASD to enhance API solubility. Herein, polymer screening would be performed in the second stage of the proposed approach by evaluating the potential of CDP146-polymer glass solutions in terms of solubility enhancement and physical stability upon storage.

As a preliminary step of screening protocol, DCM/EtOH 2:1 (*v*/*v*) was identified as the binary solvent mixture of interest for CDP146 SDSD manufacturing. The objective was to keep the same mixture of solvent during the entire duration of the screening protocol because a change in solvent system could impact the final properties of SDSD such as morphology, particle size and solid state [15,36].

### 3.2. Screening of Polymer and Stabilizer (Stage 2)

Small batches (100 mg) of CDP146 ASDs previously identified as glass solutions were produced by spray-drying. The solid state and miscibility of screened SDSDs was evaluated and results are summarized in Table 2. API-polymer systems were identified as ideal glass solutions and displayed similar T_g_ value than the respective samples produced at smaller scale. This finding confirms the results obtained during the first stage of the screening strategy and therefore controls the validity of our approach.

Elimination of the process variability factor linked to the choice of the preparation method was achieved by implementing the same manufacturing process (i.e., spray drying) at various scales. This presents the main advantage of our current strategy compared to standard screening methodologies because the generation of ‘false negatives and positives’ results during the screening phases is minimized and thereby the risk of inappropriate carrier and DL selection is reduced. Analytical results of screened ASD batches produced at 20 and 100 mg scale confirmed that the properties of the produced SDSD are scale-independent.

**Figure 5:**
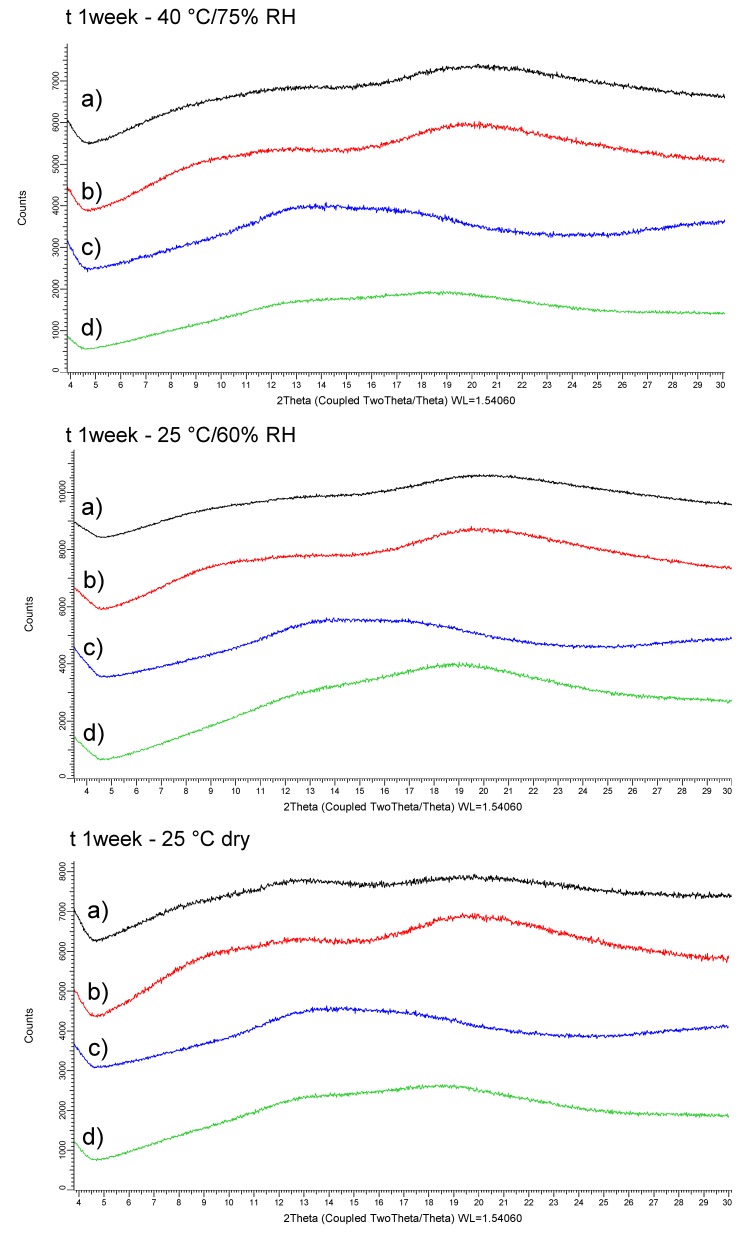
XRPD patterns of 40:60 (*w*/*w*) CDP146 SDSDs (100 mg) made with HPMCP HP50 (a), HPMCAS-LF (b), Eudragit L100 (c) and Eudragit L100-55 (d) after 1 week under stress and standard storage conditions.

The physical stability of the four CDP146 SDSDs was investigated up to one week under stress and ambient storage conditions. XRPD patterns of 40:60 (*w*/*w*) SDSDs of CDP146 stored during one week at 40 °C/75% RH, 25 °C/60% RH and 25 °C dry storage conditions, respectively, are depicted in Figure 5. No evidence of drug recrystallization was reported for the four API-polymer systems upon storage. The absence of both Bragg peaks in XRPD patterns and birefractive crystallites under polarized light observations reveal that the four screened SDSDs were found to maintain their complete amorphous state.

The four screened polymers i.e., HPMCP HP50, HPMCAS-LF, Eudragit L100 and Eudragit L100-55 were found to have similar potential to inhibit drug crystallization at solid state. This observation correlates well with the relatively high T_g_ value obtained for 40:60 (*w*/*w*) CDP146 SDSDs, detailed in Table 3. Solid dispersion with high T_g_ generate anti-plasticization effect that reduces the molecular mobility and therefore contributes to the drug stabilization in the amorphous state [37]. At this stage, the assessment of carrier’s potential to maintain amorphous state upon storage did not allow discriminating the four carriers. The selection of lead excipient would be conducted based on the dissolution performance of CDP146-polymer systems. Although the duration of the physical stability program did not discriminate API-polymer systems, this study would ensure that solid dispersions will remain physically stable between the manufacturing and the administration phases, which corresponds to an average duration of one week. Results regarding the chemical stability of screened ASDs are not presented in the current case study but would be of interest during Stage 2 [38].

**Figure 6:**
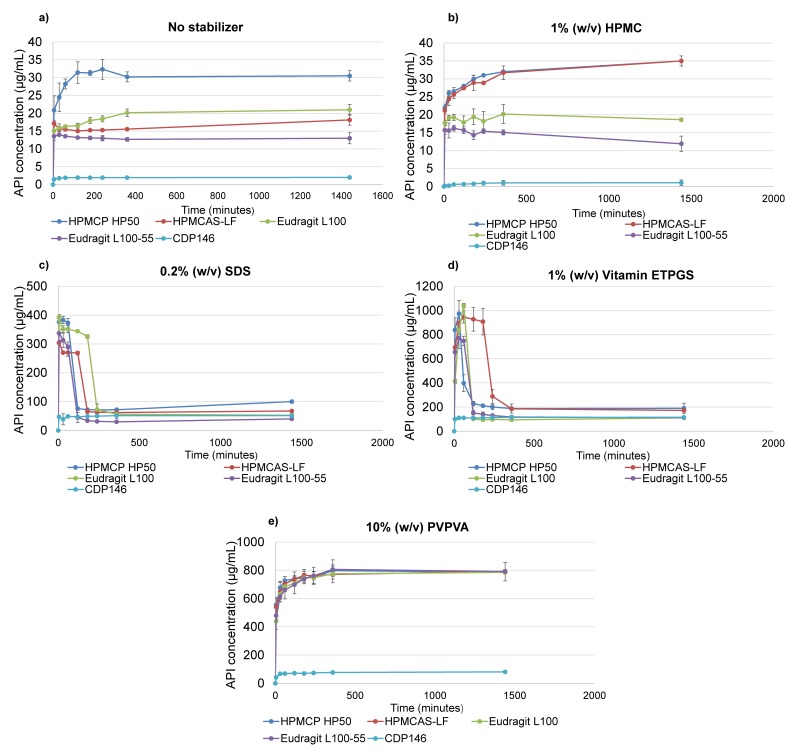
Dissolution profiles of 40:60 (*w*/*w*) CDP146 SDSDs (100 mg) and pure crystalline API in 50 mM phosphate buffer (pH 6.5) without stabilizer (**a**) or with 1% (*w*/*v*) HPMC (**b**), 0.2% (*w*/*v*) SDS (**c**), 1% (*w*/*v*) Vitamin ETPGS (**d**) and 10% (*w*/*v*) PVPVA (**e**) at an API concentration of 1 mg/mL.

Dissolution performance of API-polymer systems was evaluated at 37 °C in dissolution medium at pH 6.5 with and without stabilizers. At this stage, the generation of supersaturated solution with solubility improvement compared to crystalline drug during a minimum of 4 h is required to maximize in-vivo exposure. This length of time corresponds to the maximum duration of the administration phase during preclinical tests, typically. Dissolution profiles of screened 40:60 (*w*/*w*) CDP146 SDSDs in various media are depicted in Figure 6.

As seen in Figure 6a, the four screened ASDs showed a poor solubility enhancement compared to crystalline API which is probably explained by a sudden drug recrystallization in the first seconds of the dissolution testing. This indicates that the presence of a polymer is insufficient and addition of a stabilizer is needed to stabilize the supersaturated solution. Similarly in the presence of 1% (*w*/*v*) HPMC (Figure 6b), the dissolution profiles of the four tested SDSDs did not allow generating supersaturation and improving drug solubility, significantly. This invalidates the selection of HPMC as anti-nucleation/stabilizing agent for SDSDs of CDP146.

When adding 0.2% (*w*/*v*) SDS or 1% (*w*/*v*) Vitamin ETPGS in the dissolution medium, the dissolution profiles of the four screened SDSDs of CDP146 were found to generate supersaturation in the first minutes/hours of the tests, as seen in Figure 6c,d. The best performing API-polymer combinations include HPMCAS-LF and Eudragit L100 carriers in dissolution medium containing 1% (*w*/*v*) Vitamin ETPGS and 0.2% (*w*/*v*) SDS, respectively. These specific ASDs were found to maintain supersaturation up to 3 h corresponding to a solubility improvement of around 720% and 561% after 3 h, respectively. However, recrystallization during dissolution testing characterized by sudden drop in solubility was recorded for all ASDs, as seen in Figure 6c,d. Consequently, the stabilizing potential of SDS and Vitamin ETPGS is not enough to cover the administration phase and alternative stabilizers need to be considered.

Finally, dissolution profiles obtained for all 40:60 (*w*/*w*) CDP146 SDSDs in the medium containing 10% (*w*/*v*) PVPVA allowed generating and maintaining supersaturation and parachute effect up to 24 h, corresponding to a solubility improvement percentage of around 1000% compared to the crystalline drug. Therefore, PVPVA was found as the best performing stabilizer that allowed sustaining supersaturation generated by 40:60 (*w*/*w*) CDP146 SDSDs during a length of time that covers the administration phase. Regarding the selection of adequate carrier, the four screened SDSDs of CDP146 display similar dissolution profiles in the dissolution medium made with 10% (*w*/*v*) PVPVA and could be selected, independently. Nevertheless, results obtained in the vehicle made with 1% (*w*/*v*) Vitamin ETPGS can be considered as discriminative conditions and confirm that CDP146/HPMCAS-LF 40:60 (*w*/*w*) was the only API-polymer combination that reached drug solubility above 900 µg/mL up to 180 min, as seen in Figure 6d. All other ASDs recrystallize during dissolution test before 60 min and lost their solubility enhancement potential, consequently. HPMCAS-LF was found to have a greater potential than other tested carriers in terms of the degree of supersaturation generated and the extent of supersaturation maintenance. Based on these considerations, HPMCAS-LF was selected as adequate carrier for the manufacturing of CDP146 SDSD.

Furthermore, in the case where the interplay between carrier and stabilizer does not allow maintaining supersaturation during a sufficient time to cover the administration phase, two alternatives can be considered: the potential of other stabilizers alone or in combination (e.g., Tween 80, PEG, Soluplus, PVPK15, Docusate, cellulose derivatives…) can be evaluated and/or considering SDSD with lower DL (e.g., 30% (*w*/*w*)) at this stage of the screening approach. Decreasing the DL of solid dispersions is generally known to ease the stabilization of supersaturated solution [18].

### 3.3. Oral Formulation Development (Stage 3)

A 1 g batch of CDP146/HPMCAS-LF 40:60 (*w*/*w*), previously identified as lead API-polymer system was produced by spray-drying. The solid dispersion was characterized in terms of solid state, miscibility and residual solvent content. Results are summarized in Table 3. This SDSD displays a single T_g_ of around 102 °C in the reverse heat flow of mDSC and the absence of melting and recrystallization process in the non-reverse and total heat flow signals. Large amorphous halo in XRPD pattern confirms the complete drug amorphization after processing. The results obtained in Table 3 correlate well with the properties of CDP146/HPMCAS-LF 40:60 (*w*/*w*) generated during the first two stages of the screening approach. This confirms the advantage of the proposed screening strategy compared to standard screening methodologies because limited scale-up effects are observed with this approach and the same manufacturing technology is used during screening and manufacturing stages reducing the need for additional formulation/process development.

Additionally, the physical stability of the lead SDSD of CDP146 was assessed up to three months at 40 °C/75% RH, 40 °C dry storage conditions and 25 °C/60% RH. Results from stability study are summarized in Table 3. Under both ambient and stress conditions, CDP146/HPMCAS-LF was found to maintain complete drug amorphous state during the entire duration of the stability program, which is a good indicator for the long-term stability of the spray-dried material. This helps to gain insight into the shelf life of the product and allows covering drug development up to GLP toxicology studies [8]. Evaluation of long-term stability using Atomic force microscopy (AFM) at this stage of the formulation development can help in the reduction of stability program from months to hours, by detecting phase separation in solid dispersions systems at nanometer scale [39].

**Figure 7:**
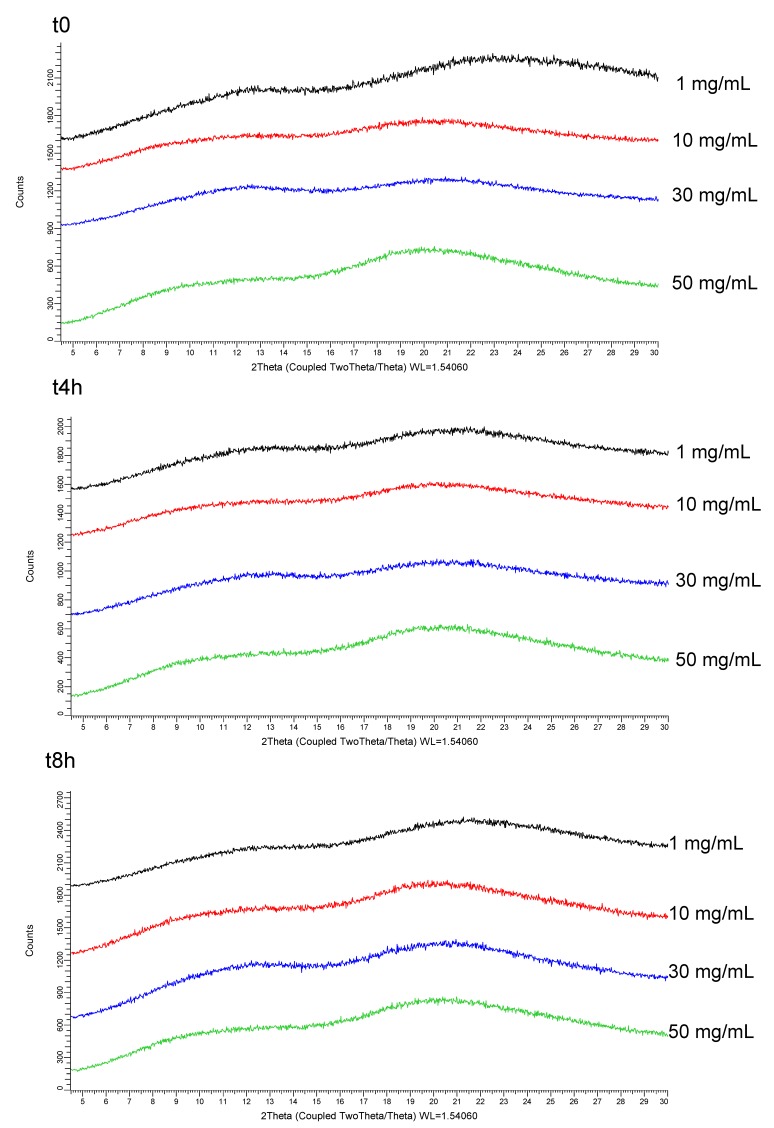
XRPD patterns of the wet solid filtrate collected from the suspension of CDP146/HPMCAS-LF 40:60 (*w*/*w*) in 10% (*w*/*v*) PVPVA, 1% (*w*/*v*) HPC-SSL and 0.1% (*w*/*v*) antifoam in 100 mM citrate buffer (pH 4) at 1, 10, 30 and 50 mg/mL.

In the scope of preclinical studies, lead SDSD of CDP146 was prepared as suspension in a liquid vehicle that contains the stabilizer agent of interest identified during Stage 2 (i.e., 10% (*w*/*v*) PVPVA) combined with 1% (*w*/*v*) HPC-SSL as a standard suspending agent and 0.1% (*w*/*v*) antifoam in 100 mM citrate buffer (pH 4). The influence of the API dose on the stability of the oral liquid formulation was assessed at 1, 10, 30 and 50 mg/mL during 8 h by monitoring drug crystallization in the formulation. The assessment of suspensions stability is a mandatory step before oral administration to animals in order to ensure that no physical change of the ASD has occurred as it can negatively impact drug exposure and lead to misleading interpretation of in-vivo results. XRPD patterns of the wet solid filtrate collected from the oral formulation at t_0_, t_4h_ and t_8h_, are depicted in Figure 7. Results obtained in XRPD reveal that the ASD in suspension remained amorphous in the vehicle and did not convert into its original crystalline state even after 8 h at an API concentration of 50 mg/mL. Similar results were obtained in XRPD after evaporation of the wet solid filtrate, while PLM observations confirm the lack of birefringence of the ASD suspension (data not shown). 

**Figure 8:**
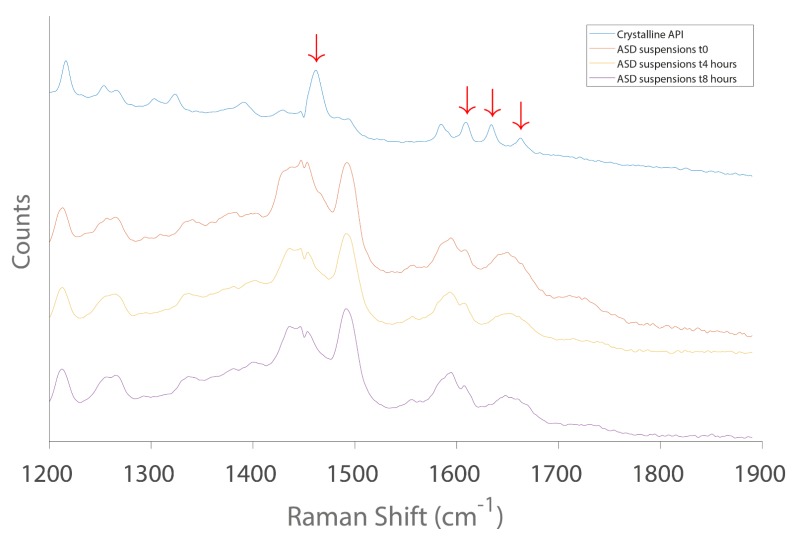
Raman spectra of pure crystalline CDP146 and CDP146/HPMCAS-LF 40:60 (*w*/*w*) suspensions (10 mg/mL) recorded at t_0_, t_4h_ and t_8h_.

Figure 8 depicted the Raman spectra of crystalline CDP146 and ASD suspension of CDP146/HPMCAS-LF 40:60 (*w*/*w*) prepared at 10 mg/mL and acquired after 0, 4 and 8 h under magnetic stirring (350 rpm). As seen in Figure 8, arrows represented in the Raman spectrum of crystalline API point the characteristic peaks of crystalline CDP146 that displayed significant difference from its amorphous counterpart in a selected region of the Raman Shift. Moreover, the Raman spectra of ASD suspension obtained after 4 and 8 h in the formulation vehicle appear to be identical to the freshly prepared formulation. As no specific peak changes were observed during the run, it is likely that the ASD suspensions remained in the amorphous state throughout the studied time. This provides evidence of the formulation capacity to remain amorphous up to 8 h and confirms the previous results obtained in XRPD and PLM. The aforementioned considerations regarding the stability of the oral formulation confirm the choices made with respect to carrier selection, DL and stabilizer used in the liquid vehicle. Although Raman spectroscopy was used to double-check the results obtained from XRPD and therefore confirms the formulation stability, its application might be optional in order to speed up the oral formulation development process. 

**Figure 9:**
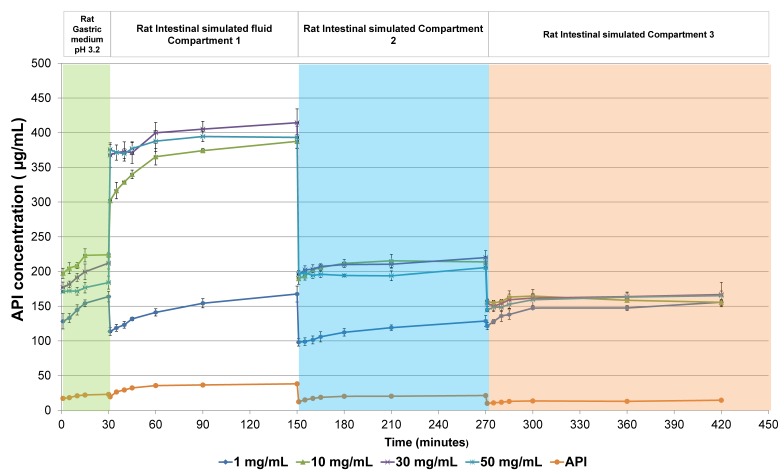
Dissolutions profiles of CDP146/HPMCAS-LF 40:60 (*w*/*w*) suspension and pure crystalline API in bio-predictive conditions mimicking gastro-intestinal tract of rat species at an API concentration of 1, 10, 30 and 50 mg/mL.

**Figure 10:**
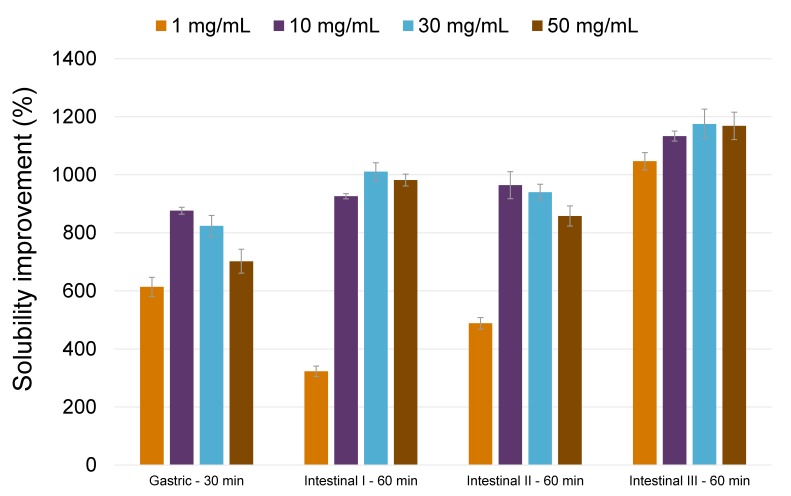
Solubility improvement (%) of CDP146/HPMCAS-LF 40:60 (*w*/*w*) suspension compared to crystalline drug after 30 and 60 min in gastric and intestinal fluids, independently.

Since the ASD suspension formulation was found to maintain its amorphous state for longer than the transit time of species used during preclinical studies, the potential of the oral formulation to enhance drug solubility in bio-predictive conditions was evaluated. In this regard, in-vitro dissolution that intended to mimic the gastro-intestinal tract of rat species was conducted as it constituted the most commonly used model during preclinical studies [8]. Dissolution profiles of crystalline drug and oral formulation prepared at various API concentrations in gastric medium and rat intestinal simulated compartments, are depicted in Figure 9. Solubility improvement percentages of oral formulation compared to crystalline drug in gastric and intestinal fluids after 30 and 60 min, respectively, are given in Figure 10. As seen in Figure 9, oral formulation prepared at four different API doses was found to enhance drug solubility, considerably in both gastric and intestinal fluids. Solubility improvement of about 1000% was obtained for oral formulation prepared at 10–50 mg/mL in intestinal compartments. The dissolution profile of each formulation displayed a plateau during the entire duration of the dissolution tests with no recrystallization process. The capacity of the tested oral formulation to maintain supersaturation in intestinal compartment of rat during the absorption window would allow enhancing oral drug bioavailability, considerably. The fact that drug did not recrystallize from supersaturated solution in gastric and first compartment of intestine can be partially attributed to the role of enteric carrier to delay drug release below the pH of polymer hydration. This confirms the great potential of using enteric polymer in the scope of preclinical activities. Despite a dilution factor of 2 between the gastric medium to the intestinal fluid, the solid dispersion formulation was found to display a ‘reservoir’ effect by recovering high drug solubility in the first minutes of dissolution in each intestinal compartment. Moreover, the different concentrations of supersaturation generated in each of the intestinal compartments, can be attributed to the different compositions of bile salts. As an example, the level of drug concentration reached by the 50 mg/mL formulation was reduced by a factor 2 between the first two intestinal compartments when SIF content was decreased from 35.6 mM to 14 mM. Additionally, no major difference was obtained in the dissolution profile of formulation prepared at 10–50 mg/mL. This can be explained on the basis that at 10 mg/mL, the amorphous solubility is reached in each of the biofluids and increasing API dose to 50 mg/mL cannot exceed this value. Further results generated during in-vivo studies would be necessary to compare the potential of 10–50 mg/mL formulations. Additional tests including drug absorption simulation by a biphasic system can be examined to discriminate formulation prepared at various doses [13]. At this stage, the conduction of dissolution tests in bio-predictive conditions reveals that oral formulation in the API dose range of 1–50 mg/mL was found to fulfill the necessary requirements to be addressed during in-vivo administration studies. In this regard, larger batch production of CDP146/HPMCAS-LF 40:60 (*w*/*w*) can be performed to support the preclinical studies.

This novel screening approach developed internally at UCB Pharma has been found to provide a rational selection of polymer and DL prior to the development and manufacturing of SDSD. The proposed three-stage decision protocol has been successfully applied in several projects and respects the development constraints in terms of API consumption and time resources. A maximum amount of 735 mg of API and a duration of 9 days are required in order to screen four polymers and four stabilizers at two different DLs. In the current case study, 600 mg of CDP146 and 9 days were needed from solvent screening stage to the oral formulation optimization.

Additionally, during the traditional ASD pathway development, once the lead API-polymer system has been identified, an additional step including process development using mini spray-dryer is required to finetune optimal DL and identify robust processing and formulation conditions for the manufacturing of SDSD. This transfer stage from screening to mini spray-dryer is basically based on a ‘trial and error’ approach and requires significant investment in time and API. In a recent study, Wyttenbach et al. (2013) estimated that the traditional ASD solvent casting program using rotary evaporator requires 16 weeks of development time and up to 10 g of API to screen 5 polymers at 2 DLs before the first ASD batch production can be started using mini spray-dryer [18]. In contrast to that, the implementation of spray-drying in a small-scale approach as it occurs for the first time in the proposed strategy allows removing this supplementary stage in ASD development. Herein, the downscaling approach proposed in the current study constituted a more simple and efficient methodology than traditional formulation development, reducing API consumption by a factor 13 and screening/formulation development time by a factor 12.

Contrary to classical ASD development using film casting or quench cooling during the screening phase, the proposed strategy allowed reducing the influence of the preparation method on the polymer and DL selection. Based on experience, it is almost impossible to change the carrier identified during screening phases once the manufacturing phase has started. Results obtained in the current study highlighted that small-scale batches of SDSD generated in the first stage of the screening strategy display similar properties in terms of miscibility, T_g_ value and solid state compared to larger scale production. This allows to finetune DL selection in the first stage of screening stage and gain insight into the final performance of SDSD.

Particular attention has been paid to propose a screening strategy where only standard analytical equipment were needed, to ensure this approach can be simple enough to be applicable in various pharmaceutical development laboratories. To enable solid state and miscibility characterization of screened SDSDs before and after incubation, the use of mDSC, XRPD and PLM was combined. Non-sink dissolutions conditions with regard to the crystalline API were performed to assess the solubility enhancement potential of screened SDSDs and the extent of supersaturation.

Although drug–polymer interactions have not been investigated, the authors assume that the results obtained during the physical stability studies and dissolution tests provide valuable information on the polymer’s potential to interact with the drug by preventing recrystallization [40]. Additionally, the systematic evaluation of drug–polymer miscibility has given insight into solid dispersion performance and homogeneity i.e., the formation of ideal glass solution is a proof of drug–polymer homogeneity at molecular level. Further studies will focus on adapting this screening approach for the development of ternary SDSDs in which a second polymer/surfactant is added to the ASD blend to improve the solid state stabilization of the amorphous drug as well as the maintenance of supersaturation during dissolution [41,42].

## 4. Conclusions

The proposed screening approach implements for the first time spray-drying in a methodical small-scale approach for the development of ASD during preclinical activities. This novel screening strategy based on a three-stage decision protocol was verified with CDP146 by evaluating the performance of SDSDs in terms of drug–polymer miscibility, physical stability and in-vitro dissolution. Among the four polymers screened, HPMCAS-LF was found as the adequate carrier to provide physically stable SDSD of CDP146 at 40% (*w*/*w*) DL. Best performing stabilizer (10% (*w*/*v*) PVPVA) was identified during Stage 2 of the proposed strategy, to help maintain supersaturation during the absorption window of orally administrated suspension formulations.

The total duration of the screening and the oral formulation development phases require 9 days and a maximum of 735 mg of API to screen four polymers and four stabilizers at two different DLs. In the current study where the screening protocol was verified with CDP146, only 600 mg of CDP146 and 9 days were needed. In this regard, the proposed screening approach can be classified as a material sparing approach, particularly adapted for the development of SDSDs in the industrial sector. Moreover, the choice of using only standard analytical equipment (e.g., mDSC, XRPD, PLM and TGA) during the screening protocol would ensure wide applicability and facilitate its use to a large number of development groups in the pharmaceutical industry.

Small ASD batches as low as 20 mg were obtained by spray-drying and were representative from larger scale SDSD productions in terms of drug–polymer miscibility and solid state. To this extent, this downscaling approach has never been reported in literature, previously and would allow reducing efforts to correlate information from bench to batch manufacturing. Compared to standard screening methodologies e.g., solvent casting and quench cooling, the proposed screening strategy would improve the prediction accuracy with regards to SDSD properties and performance, resulting in a de-risking and rational selection of appropriate carrier and DL. This is explained on the basis that the process variability factor linked to the choice of the preparation method is minimized. Application of this novel and superior screening approach in UCB projects has replaced previous practices as it demonstrated a straight pathway from screening to manufacturing phases and eased the drug development progress, considerably.

## Figures and Tables

**Table 1:** Physico-chemical properties of screened polymers for CDP146 ASD development.

Polymer	M_w_ (g/mol) ^a^	Dissolution pH ^a^	T_g_ (°C) ^b^	T Degradation (°C) ^c^
HPMCP-HP50	78,000	>5.0	140	160
HPMCAS-LF	18,167	>5.5	122	170
Eudragit L100	125,000	>6.0	192	165
Eudragit L100-55	320,000	>5.5	122	165

^a^ data obtained from available literature [22]; ^b^ data experimentally obtained by modulated differential scanning calorimetry (mDSC); ^c^ data experimentally obtained by thermogravimetric analysis (TGA).

**Table 2:** Characterization summary of 40:60 CDP146 ASDs produced by spray-drying (100 mg).

ASD Composition		Yield	Miscibility/Solid State		
Polymer	DL (*w*/*w*)	Yield (%)	T_g_ (°C)	T_m_ (°C)	XRPD Pattern/PLM
HPMCP HP50	40%	68.1	113.5	-	A
HPMCAS-LF	40%	70.9	102.5	-	A
Eudragit L100	40%	71.8	163.4	-	A
Eudragit L100-55	40%	71.7	120.9	-	A

A: amorphous sample characterized by the presence of a large halo in XRPD and the absence of birefringence under PLM observations.

**Table 3:** Characterization summary and physical stability evaluation of lead CDP146 SDSD produced by spray-drying (1 g).

Lead ASD		Process Considerations	Miscibility/Solid State			Residual Solvent	Physical Stability								
Polymer	DL (*w*/*w*)	Yield (%)	T_g_ (°C)	Tm (°C)	XRPDPattern/PLM	Weight Loss (%)	25 °C/60% RH			40 °C Dry			40 °C/75% RH		
							1 m	2 m	3 m	1 m	2 m	3 m	1 m	2 m	3 m
HPMCAS-LF	40%	88.2	102.5	-	A	0.2	A	A	A	A	A	A	A	A	A

A: amorphous sample characterized by the presence of a large halo in XRPD and the absence of birefringence under PLM observations.

## Abbreviations

ACN	Acetonitrile
AFM	Atomic force microscopy
API	Active pharmaceutical ingredient
ASD	amorphous solid dispersions
DCM	Dichloromethane
DL	Drug-loading
EtOH	Ethanol
HPLC	High performance liquid chromatography
mDSC	Modulated differential scanning calorimetry
NCE	New chemical entity
PLM	Polarized light microscopy
RH	Relative humidity
SDSD	Spray-dried solid dispersion
SIF	Simulated intestinal fluids
T_g_	Glass transition temperature
TGA	Thermogravimetric analysis
T_m_	Melting temperature
XRPD	X-ray powder diffraction
